# A Case of Panic Attacks Developing After 10 Years of Chronic Cannabis Use in a Patient With No Prior Psychiatric History

**DOI:** 10.7759/cureus.34197

**Published:** 2023-01-25

**Authors:** Grace A Johnson, Lucy Guerra, Asa Oxner

**Affiliations:** 1 College of Medicine, University of South Florida Morsani College of Medicine, Tampa, USA; 2 Department of Internal Medicine, University of South Florida Morsani College of Medicine, Tampa, USA

**Keywords:** cannabis (marijuana), drug addiction, heart palpitations, marijuana abuse, agoraphobia, generalised anxiety disorder, panic disorders, cannabis use disorder

## Abstract

Cannabis use for medical and recreational purposes is increasing. Inhibitory activity of cannabinoids (CB) at the CB1 and CB2 receptors centrally and peripherally mediate the therapeutic effects that are wielded for palliation of pain, anxiety, inflammation, and nausea in indicated conditions. Cannabis dependence is also associated with anxiety; however, the direction of causality is unknown, such as whether anxiety disorders lead to cannabis use, or whether cannabis contributes to the development of anxiety disorder. The evidence hints that both may have validity. Here we present a case of cannabis-associated panic attacks following 10 years of chronic cannabis dependence in an individual with no prior psychiatric history. The patient is a 32-year-old male with no significant past medical history who presented complaining of five-minute episodes of palpitations, dyspnea, upper extremity paresthesia, subjective tachycardia, and cold diaphoresis occurring in a variety of circumstances for the past two years. His social history was significant for 10 years of smoking marijuana multiple times daily, which he had quit over two years ago. The patient denied past psychiatric history or known anxiety problems. Symptoms were unrelated to activity and only relieved with deep breathing. The episodes were not associated with chest pain, syncope, headache, or emotional triggers. The patient had no family history of cardiac disease or sudden death. The episodes were refractory to the elimination of caffeine, alcohol, or other sugary beverages. The patient had already stopped smoking marijuana when the episodes began. Due to the unpredictable nature of the episodes, the patient reported a growing fear of being in public. On laboratory workup, metabolic and blood panels were within normal limits, as well as thyroid studies. Electrocardiogram showed normal sinus rhythm, and continuous cardiac monitoring revealed no arrhythmias or abnormalities despite the patient indicating multiple triggered events within the duration of monitoring. Echocardiography also showed no abnormalities. With organic cardiac causes of the subjective palpitation episodes ruled out, a psychogenic etiology of the episodes was presumed, and the patient was referred to behavioral health services. In conclusion, cannabis-induced anxiety or panic disorders should be considered in patients with no prior psychiatric history presenting with anxiety-like attacks following a period of cannabis dependence or current use. These patients should be advised to cease cannabis use and referred to behavioral medicine.

## Introduction

Cannabis use among young adults has significantly increased in the past 10 years, from 29% of 19 to 30 year-olds in 2011 reporting past-year marijuana use, to 43% reporting such use in 2021 [1]. Medical marijuana has been legalized in most of the United States for palliation of neurodegenerative diseases such as multiple sclerosis, Parkinson’s disease and amyotrophic lateral sclerosis, cancer or chronic pain, HIV/AIDS, post-traumatic stress disorder (PTSD), and other chronic diseases such as Crohn's disease, epilepsy, and glaucoma [2]. However, cannabis dependence is known to cause adverse effects such as memory impairment [3], decreased intelligence quotient (IQ) in individuals who used heavily during adolescence [4], and inferior social outcomes such as poor work commitment and financial instability [5]. A more recently recognized adverse effect in a subset of users is cannabinoid hyperemesis syndrome [6,7]. Whether palliative or pathological, the effects of cannabis use are primarily mediated by delta-9-tetrahydrocannabidiol (THC) on CB1 and CB2 receptors, which are G-protein coupled receptors that inhibit adenylyl cyclase to decrease levels of cAMP [7] and have a wide array of physiologic effects. For example, THC at the dorsal vagal complex and the intestinal epithelium is anti-emetic [7-10], whereas the metabolite cannabigerol is shown to be a CB1 and 5-HT1A antagonist, which may be responsible for the emetogenic effect of cannabis in some individuals [7].

Cannabis use is also associated with anxiety disorders [11]. The general prevalence of panic disorder and general anxiety disorder in the United States is 2.7% and 3.1%, respectively [12]. Anxiety and panic disorders are seen to exist in a higher proportion in individuals with substance use disorders, with studies indicating a two-fold increased risk of lifetime panic attacks in association with alcohol dependence [13] or psychedelic abuse [14]. While cannabis in particular may be used by individuals with anxiety disorders to palliate their symptoms [15,16], there may also be an increased risk of anxiety disorders as a consequence of long-term cannabis use [11,17]. Overall there is a significant relationship between cannabis use and lifetime panic or anxiety disorders, but it is difficult to establish a causal relationship [18] due to a paucity of prospective studies - it is unclear whether panic or anxiety disorders lead to cannabis use to quell sympathetic nervous system hyperactivity subjectively, or if anxiety disorders arise from cannabis use. Here, we present the case of a patient with no prior history of panic or anxiety disorders who developed panic attacks after 10 years of chronic cannabis use despite abstinence for years before symptom onset.

## Case presentation

We present a 32-year-old male with no significant past medical history who presented for a follow-up on palpitations that began two years prior. The episodes comprised feelings of unease with subjective tachycardia, dyspnea, upper extremity paresthesia, occasionally nausea, cold sweating, and feeling "woozy" that last for approximately five minutes. These had occurred between two and four times monthly for the past two years. They would onset randomly, regardless of the social or professional circumstance, and even in the middle of the night. The patient had visited the emergency department for the episodes on previous occasions. The episodes seemed to be exacerbated by alcohol and caffeine intake and would subside with deep breathing. The patient denied associated syncope, headache, chest pain, or visual or movement disturbances, and had not been experiencing insomnia or depressive symptoms. He otherwise denied general anxious symptoms, even endorsing a sense of surprise at having these episodes as he had not considered himself an "anxious person."

His social history was significant for a past history of chronic cannabis dependence, in which he smoked cannabis multiple times daily for 10 years, but quit over two years ago. He used alcohol sparingly and socially. Other possible sources of stressors included the arrival of a new baby, however, the panic attacks had well preceded the family change, and the patient did not note an increase in frequency since the arrival of his child. He denied feeling stressed at work. He took no medications or supplements. There was no family history of sudden death, nor atherosclerotic or structural cardiac defects.

The patient came into the office after attempting lifestyle interventions to ameliorate the episodes, eliminating alcohol, caffeine, and sugary beverages from his diet, however, he still had four episodes since the changes were instituted three weeks prior. Two of the episodes occurred at home and two occurred at work. The patient reported growing feelings of wariness to leave the house to go out to dinner or park for fear of having an episode.

**Figure 1:**
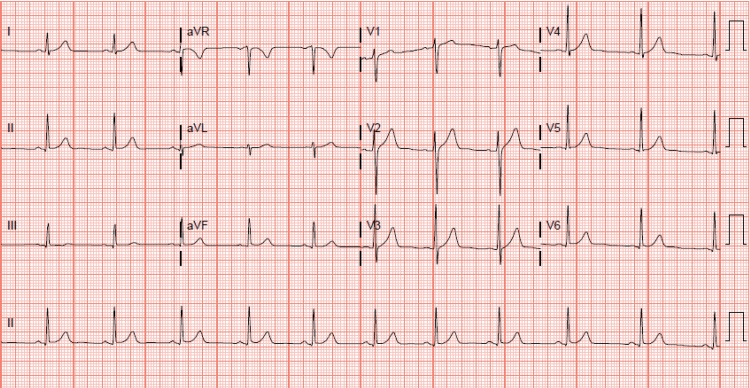
ECG at the time of presentation Electrocardiogram (ECG) shows the patient in normal sinus rhythm, with a normal axis and corrected QT interval of 414 milliseconds.

Differential diagnoses included panic disorder, anxiety disorder, structural heart defects such as hypertrophic cardiomyopathy, hyperthyroidism, and benign paroxysmal arrhythmias such as premature atrial or ventricular contractions. Upon workup, the complete metabolic panel and complete blood count were found within normal limits, as well as thyroid-stimulating hormone (TSH) and free T4. The lipid panel was notable for slight hyperlipidemia with an LDL of 153 mg/dL (Table 1). Electrocardiogram (ECG) revealed a normal sinus rhythm with a normal axis (Figure 1). In the absence of ECG abnormalities, the patient was referred for continuous cardiac monitor placement to assess for arrhythmias. Instructions were for a 14-day placement, yet it was only feasible to maintain its placement for one day due to perspiration causing dislodgement of the monitor.

**Table 1:** Labs prior to presentation. BUN: blood urea nitrogen, HDL: high-density lipoprotein, LDL: low-density lipoprotein, TSH: thyroid-stimulating hormone

Laboratory values	
Complete blood count	
White blood cells	6.5 k/uL
Hemoglobin	14.3 g/dL
Hematocrit	42.1%
Platelets	247 k/uL
Complete metabolic panel	
Sodium	140 mmol/L
Potassium	4.6 mmol/L
Bicarbonate	23 mmol/L
Chloride	101 mmol/L
BUN	14 mg/dL
Creatinine	0.9 mg/dL
Glucose	89 mg/dL
Lipid panel	
Total cholesterol	217 mg/dL
Triglycerides	98 mg/dL
HDL	46 mg/dL
LDL	153 mg/dL
Thyroid function tests	
TSH	1.5 uIU/mL
Free T4	1.15 ng/dL

Within the time recorded by the monitor, the patient’s average heart rate (HR) was 80 bpm, eight trigger events were indicated, and the HR during these triggers ranged from 83-138 bpm while remaining in normal sinus rhythm. There was no evidence of ventricular tachycardia, atrial fibrillation, pauses, heart blocks, or other arrhythmias. Echocardiography was also performed, which revealed no wall motion abnormalities, normal-sized chambers, and no valvular dysfunction. At the follow-up visit, it was recommended to continue supportive measures and consider behavioral health interventions. The patient was not interested in behavioral health support, but continued to limit caffeine and alcohol, and maintain marijuana abstinence. However, he remained to have occasional panic attacks.

## Discussion

A 1997 case series [19] recounts cannabis-associated panic or anxiety disorders in which the initial attack occurred during cannabis use, and recurrent attacks occurred after cannabis cessation. In one case, a 53-year-old female suffering from major depressive disorder accidentally ingested cannabis-laced chocolate mousse and developed a panic attack, which was then followed by daily panic attacks for three weeks; these resolved after initiation of 25mg amitriptyline nightly and 20mg citalopram every morning. The second case involved a 30-year-old male who experienced panic attacks onsetting after 10 years of cannabis use in the context of a dysthymic disorder; he was referred to a psychotherapist but was lost to follow-up. The third case involved a 35-year-old woman who smoked cannabis weekly for 15 years, and then daily for two years. After smoking daily for two years, she had a panic attack with tachycardia, diaphoresis, palpitations, and anxiety immediately after smoking. She ceased smoking after this, but the attacks recurred frequently and in various situations, causing her to develop agoraphobia. She was treated with cognitive-behavioral therapy. Zvolensky et al. [20] found that in 941 adolescents who were followed into young adulthood without pre-existing panic attacks or disorder, the odds of developing panic attacks and panic disorder was 3.7, compared to 4.9 in those with cannabis dependence. However, this association was weakened once stratified by daily cigarette smoking, suggesting a role of nicotine in augmenting anxious symptoms in this study population. A meta-analysis investigating the relationship between baseline cannabis use and future anxiety symptoms saw a slight increase in anxiety from baseline to follow-up [16]. Additionally, a 2021 meta-analysis of epidemiological surveys revealed a strong association between cannabis use disorder and generalized anxiety disorder with an odds ratio of 2.99 (2.14-4.16) [15]. While these studies do not clarify a directional relationship in the causality between cannabis use and anxiety or panic pathophysiology, there does seem to be a subset of individuals who go on to develop psychiatric issues without any preexisting history of such disturbances. This seems to be a paradoxical effect of cannabis which typically is esteemed by users for its anxiolytic, anti-emetic, or otherwise calming effects on the central nervous system. The author posits that perhaps a sensitivity to metabolites such as cannabigerol that are antagonists at inhibitory receptors is what mediates these paradoxical effects in some patients with chronic use.

Furthermore, there has been an increase in reports of hospitalizations related to the use of delta-8-THC, which is an isomer of the delta-9-THC compound typically invoked when discussing cannabis consumption. Delta-8-THC is an easily available yet dangerously potent cannabis product that is associated with acute psychosis, suicidality, anxiety, tremors, and unconsciousness, and it contains contaminants resulting from an unregulated synthesis process [21]. It will be important for physicians to be vigilant in recognizing substance-induced psychiatric symptoms associated with new variants of cannabis products, as well as educate patients about their harms. 

## Conclusions

Here we present a patient with no known prior psychiatric history of depression or anxiety who presented with seemingly idiopathic panic disorder onsetting after daily cannabis use for 10 years despite present abstinence. The conclusions from this case are strengthened by the temporality of symptom onset in suggesting that chronic cannabis use can contribute to a future anxiety disorder. However, a weakness is that despite the patient endorsing no perception of anxiety problems, it is possible the patient did indeed have an underlying anxiety disorder that was undisclosed or not formally diagnosed that could have coincidentally manifested at this time.

With increasing legalizations of both medical and recreational cannabis use, which have tempered the stigma that previously necessitated its surreptitious use, patients may be more likely to report underlying substance use that may correlate with symptoms. New and seemingly unexplained psychiatric complaints should be met with a detailed substance use history in an otherwise unrevealing history.
